# A Role of Sphingosine in the Intracellular Survival of *Neisseria gonorrhoeae*

**DOI:** 10.3389/fcimb.2020.00215

**Published:** 2020-05-12

**Authors:** Franziska Solger, Tobias C. Kunz, Julian Fink, Kerstin Paprotka, Pauline Pfister, Franziska Hagen, Fabian Schumacher, Burkhard Kleuser, Jürgen Seibel, Thomas Rudel

**Affiliations:** ^1^Chair of Microbiology, University of Würzburg Biocenter, Würzburg, Germany; ^2^Department of Organic Chemistry, University of Würzburg, Würzburg, Germany; ^3^Department of Toxicology, University of Potsdam, Nuthetal, Germany; ^4^Department of Molecular Biology, University of Duisburg-Essen, Essen, Germany

**Keywords:** *Neisseria gonorrhoeae*, sphingosine, sphingolipids, sphingosine kinases, invasion, survival, click chemistry

## Abstract

Obligate human pathogenic *Neisseria gonorrhoeae* are the second most frequent bacterial cause of sexually transmitted diseases. These bacteria invade different mucosal tissues and occasionally disseminate into the bloodstream. Invasion into epithelial cells requires the activation of host cell receptors by the formation of ceramide-rich platforms. Here, we investigated the role of sphingosine in the invasion and intracellular survival of gonococci. Sphingosine exhibited an anti-gonococcal activity *in vitro*. We used specific sphingosine analogs and click chemistry to visualize sphingosine in infected cells. Sphingosine localized to the membrane of intracellular gonococci. Inhibitor studies and the application of a sphingosine derivative indicated that increased sphingosine levels reduced the intracellular survival of gonococci. We demonstrate here, that sphingosine can target intracellular bacteria and may therefore exert a direct bactericidal effect inside cells.

## Introduction

Since the last decade, cases of bacterial sexual transmitted diseases (STDs) are rising worldwide again. Among those, gonorrhea, the second most frequent STD with 106 million cases per year (WHO), is caused by infections with the obligate human pathogen *Neisseria gonorrhoeae*. An alarming development is the current emergence of multi-drug-resistant *Neisseria* strains which led to the ranking of *N. gonorrhoeae* as a new superbug (Unemo and Shafer, 2014). First reports identified strains which are resistant against the current first-line treatment with ceftriaxone (Ohnishi et al., 2011) and azithromycin (Eyre et al., 2018).

*N. gonorrhoeae* is a gram-negative diplococcus, which infects a variety of mucosal tissues, including endocervix, pharynx, urethra, rectum and conjunctiva (Britigan et al., 1985). In rare cases, *Neisseria* is able to enter the bloodstream and cause systemic disseminated gonococcal infections (DGI) with serious consequences like endocarditis and arthritis (Lee et al., 2015). Disseminated gonococcal infection is linked to the expression of the major outer membrane protein PorB_IA_, which facilitates the invasion of gonococci through the binding of scavenger receptor expressed on endothelial cells (SREC-I) (Rechner et al., 2007). This invasion mechanism is independent of the neisserial virulence factors type IV pili and Opa (Opacity-associated) proteins, but depends on low phosphate concentrations (Zeth et al., 2013). We have previously shown that the PorB_IA_-dependent invasion leads to a re-localization of SREC-I to membrane rafts and phosphorylation of caveolin-1 via the signaling molecules phosphoinositide 3-kinase (PI3K) and phospholipase Cγ1 (PLCγ1) (Faulstich et al., 2013). The invasion process is highly dependent on intact membrane rafts, which are dynamic microdomains enriched with sphingolipids (Bieberich, 2018). Attachment and invasion of gonococci induces the accumulation of ceramide generated by the turnover of sphingomyelin (SM) through the activity of neutral sphingomyelinase (nSMase) (Faulstich et al., 2015). In contrast, the acid sphingomyelinase (aSMase) is involved in other invasion pathways of gonococci mediated by Opa-invasins (Grassmé et al., 1997) and in invasion of many other bacteria (Smith and Schuchman, 2008).

In general, sphingolipids are important membrane components for pathogens. On the one hand they can act as host cell membrane receptors, which are recognized by pathogens for adherence. On the other hand sphingolipids build together with cholesterol lipid-rafts, which serve as signaling platforms for adherence and invasion receptors (Hanada, 2005). All sphingolipids have a hydrophobic sphingoid base backbone [i.e., 2*S*,3*R*,4E-2-aminooctadec-4-ene-1,3-diol; i.g. sphingosine, sphinganine or phytosphingosine; (Futerman and Hannun, 2004)] containing a hydrocarbon chain, an amine group and two hydroxyl groups in common. The amine group is linked to a fatty acid (varying in chain length and degree of saturation) and one of the hydroxyl groups can be functionalized to a phosphate, phosphocholine, or carbohydrate. Key molecules of sphingolipid signaling are sphingosine-1-phosphate (S1P), sphingosine and ceramide, which are involved in very diverse cell processes like proliferation, differentiation, senescence, necrosis and apoptosis (Bartke and Hannun, 2009). Ceramide also serves as branch point of the metabolic pathway (Pralhada Rao et al., 2013) and can be converted to sphingosine through the action of ceramidase. Sphingosine is metabolized further by phosphorylation through an ATP-dependent sphingosine kinase (SphK), resulting in S1P (Olivera et al., 1999). An antimicrobial activity of sphingolipids is described for gram-positive and gram-negative bacteria (Fischer et al., 2012), protozoan parasites (Denny et al., 2006), enveloped viruses (Sakamoto et al., 2005) and fungi (Rollin-Pinheiro et al., 2016). In this study, we focused on the downstream signaling pathway of ceramide in the PorB_IA_-dependent invasion and intracellular survival of *Neisseria*. We show that sphingosine generated by ceramide downstream signaling pathways plays a major role in the survival of intracellular gonococci. Sphingosine taken-up from the host cell is incorporated into the bacterial membrane. Further, our data suggest that sphingosine recruited to intracellular gonococci directly inhibits gonococcal growth and leads to efficient killing.

## Results

### Sphingosine Kinases Are Involved in Gonococcal Infection

**Figure 1:**
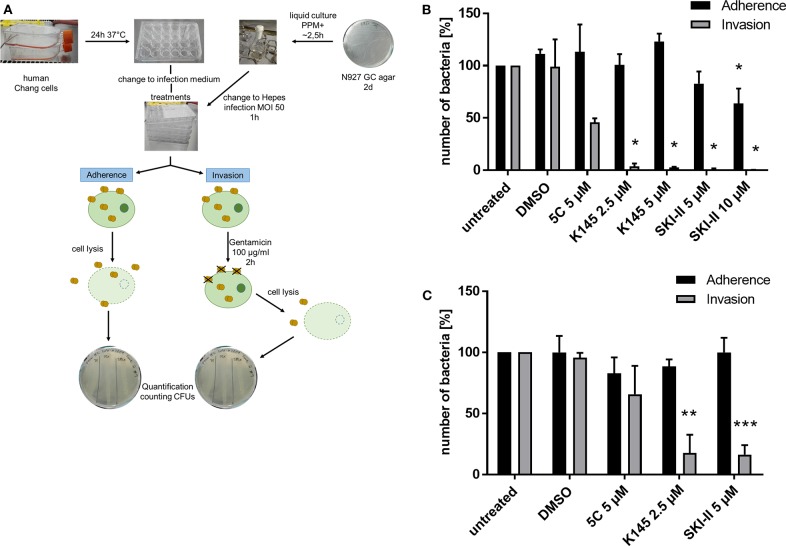
Effects of SphKs inhibitors on intra- and extracellular *Neisseria*. **(A)** Scheme of Gentamicin protection assay with *N. gonorrhoeae*. **(B)** Effect of SphK inhibitors on the infection of Chang cells with N927. The SphK inhibitors were added to Chang cells at the indicated concentrations for 2 h before infection with N927 as outlined in **(A)**. **(C)** The experiment described in **(B)** was repeated with End1 cells. Shown is the mean ± SD of three independent experiments. *p < 0.05, **p < 0.001, ***p < 0.0001 student's *t*-test relative to untreated cells.

**Figure 2:**
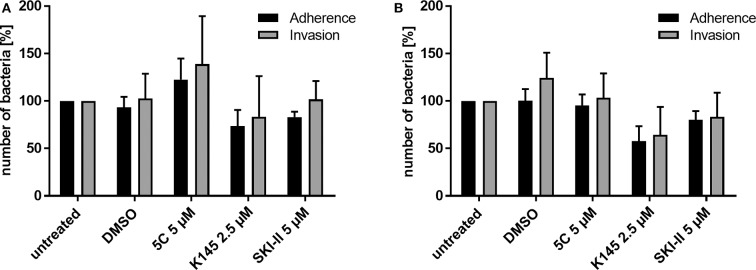
Differential immunofluorescence staining to distinguish between extra- (adherent) and intracellular (invasive) *Neisseria* N927 in Chang cells. Treatment with SphK inhibitors and infection with N927 was performed as for the gentamicin protection assay. First, extracellular bacteria were immunolabeled with anti-gonococcal antibody and secondary antibody Cy5, both incubated for 1 h each, before permeabilization of the cells with 0.1% Triton for 15 min and blocking with BSA. Then, a second anti-gonococcal antibody was added for 1 h and labeled with Cy2 conjugated secondary antibody for another 1 h. The quantification of the immunofluorescence was done either by manually counting **(A)** or by using an automated microscope (Operetta) system **(B)**. Error bars show the mean ± SD of three independent experiments.

Previously we showed that PorB_IA_-dependent invasion of *N. gonorrhoeae* requires sphingolipid-rich membrane rafts (Faulstich et al., 2015). The interaction of PorB_IA_ with SREC-I introduces changes in the sphingolipid composition of these membrane rafts, which involves the activity of nSMase. Epithelial cells infected with PorB_IA_-expressing gonococci display accumulations of ceramide on their surface (Faulstich et al., 2015). To investigate, how downstream signaling events affect bacterial invasion, we investigated the role of sphingosine kinases (SphKs) on neisserial adherence and invasion with the laboratory strain N927 (Figures 1, 2) and the clinical isolate 24871 (Zeth et al., 2013) (Supplementary Figure 1). To this end, gentamicin protection assays were performed in cells pre-treated with SphK inhibitors (Figure 1A). The chosen inhibitors exhibit a specificity against one or both kinases like 5C for inhibition of SphK1 (Wong et al., 2009), K145 for SphK2 (Liu et al., 2013) and SKI-II for both, SphK1 and SphK2 (French et al., 2003). Because cytotoxic effects of these inhibitors were already reported (Liu et al., 2013), different concentrations of these chemicals were tested on neisserial growth and cellular apoptosis to choose sub-toxic concentration for each inhibitor (Supplementary Figures 2–5). SKI-II at the concentrations used inhibited neisserial growth in liquid culture (Supplementary Figure 5A), but had no adverse effect on the adherence of bacteria compared to control cells (Figures 1, 2). Moreover, we examined whether inhibition of SphKs in Chang cells is accompanied by an alteration of *de novo* formed dihydrosphingosine and sphingosine levels as these molecules are the physiological substrates of SphKs. It is of interest that modulation of the monitored long-chain bases was dependent on the SphK inhibitors used. While inhibition of SphK1 via 5C did not result in alterations of the sphingoid bases, the application of the SphK2 inhibitor (K145) or the SphK1/2 inhibitor (SKI-II) led to a dose-dependent increase of dihydrosphingosine and sphingosine (Supplementary Figure 6). Chang (Figure 1B) and End1 cells (Figure 1C) were pretreated with these inhibitors or the solvent DMSO and infected with N927. For both cell lines a similar pattern of adherent and invasive bacteria, compared to the respective untreated control, could be detected. The adherence of *Neisseria* was not affected by blocking SphKs. Only at the highest concentration of SKI-II (10 μM), a slight decrease in adherence was detectable, probably due to a toxic effect of the inhibitor on *Neisseria* (Supplementary Figure 5A). In contrast, all inhibitors drastically reduced the number of invasive bacteria (Figures 1B,C). The weakest effect could be seen for 5 μM 5C, with a reduction of about 50% and 25% in Chang and End1 cells, respectively. This assay was repeated with the clinical isolate 24871 in Chang cells (Supplementary Figure 1A). To reduce a toxic effect of the inhibitor SKI-II on this strain, the concentration had to be reduced to 2.5 μM (Supplementary Figure 7). All three inhibitors reduced adherence of 24871. Like for N927, inhibition of SphK2, but not SphK1 significantly reduced invasion of this strain. SKI-II treatment at 2.5 μM did not affect intracellular gonococci of 24871 in line with a strongly reduced effect of these conditions on sphingosine levels (Supplementary Figure 6). Taken together, these findings suggest that SphKs are relevant for intracellular gonococci and SphK2 has a more prominent role than SphK1.

### Sphingosine Kinases Play a Role in Intracellular Survival of Gonococci

The gentamicin protection assay used to determine the effect of SphK inhibitors on intracellular gonococci does not allow to discriminate between the uptake (invasion) and intracellular survival of bacteria. The results of the inhibitor studies and the drastic reduction of recovered CFU of intracellular *N. gonorrhoeae* could therefore be due to a reduced invasion or impaired survival of invasive gonococci. To address this question, we performed differential immunofluorescence staining which allowed us to distinguish between extra- (adherent) and intracellular (invasive) bacteria irrespective of the survival of the bacteria. Since discrimination of intra- and extracellular bacteria with this method is challenging, we decided to quantify the results manually (Figure 2A) and by utilizing an automated fluorescence microscope (Operetta, Figure 2B). The results of the adherence assays were comparable to those obtained with the gentamicin protection assay for both strains N927 and 24871. However, in contrast to the results of the gentamicin protection assay, intracellular gonococci were not reduced upon SphK inhibition (Figure 2), indicating that SphKs affect gonococcal survival but not invasion in this assay.

### Sphingosine Is Toxic for Gonococci

**Figure 3:**
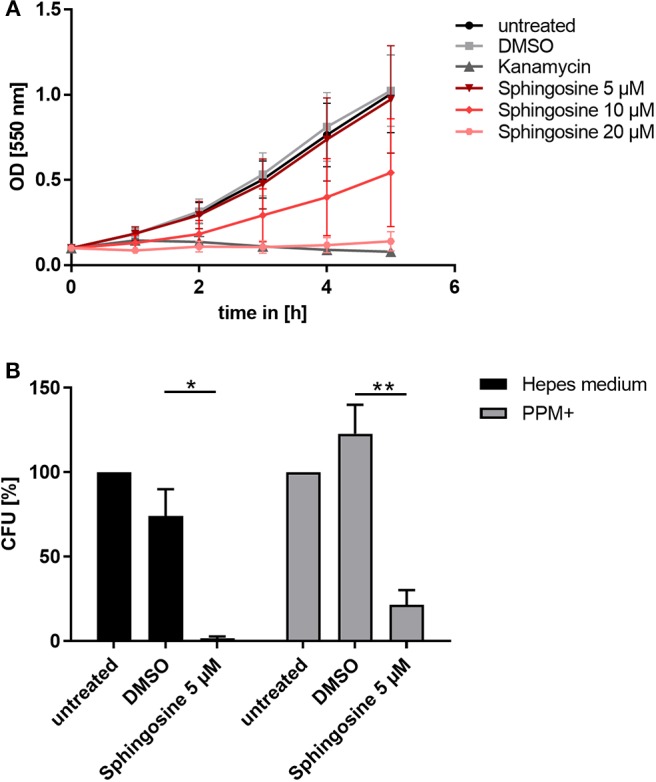
Toxic effect of sphingosine on *N. gonorrhoeae* N927. **(A)** Effect of different sphingosine concentrations on N927 *in vitro*. **(B)** Liquid cultures of N927 in Hepes medium or PPM+ were incubated with 5 μM sphingosine or DMSO as solvent control for 2 h and dilutions were then plated on GC agar plates. Plates were incubated for 24 h and CFUs were counted. Error bars show the mean ± SD of three independent experiments. *p < 0.05, **p < 0.001 student's *t*-test relative to untreated cells.

Since inhibition of SphK affected survival of intracellular gonococci, we considered the misbalanced sphingolipid components as a possible reason. A potent antimicrobial effect of sphingolipid metabolites on different bacterial species in liquid culture has been described before (Fischer et al., 2012; Becam et al., 2017). The alterations of sphingosine levels due to the different SphK inhibitors correlated very well with the measured parameters of gonococcal infection. Therefore, we considered that increased levels of sphingosine are responsible for the attenuated bacterial infection. To prove this hypothesis, the impact of sphingosine on neisserial growth was tested by performing growth curves in liquid culture for the laboratory strains N927 (Figure 3A) and FA1090 (Supplementary Figure 8A) and the clinical isolates 24871 (Supplementary Figures 8B,C) and VP1 (Supplementary Figure 8D). At the highest tested concentration (20 μM), sphingosine had a similar toxic effect on N927 gonococci as the treatment of the bacteria with the antibiotics kanamycin. The second highest dose (10 μM) caused a reduction in neisserial growth, whereas 5 μM had no effect on the replication over the incubation time of 5 h. Strain FA1090 and the clinical isolates were more resistant to sphingosine treatment than N927 but inhibition of growth in the presence of sphingosine could be demonstrated for all of them (Supplementary Figure 8).

To investigate if sub-inhibitory concentrations of sphingosine affect gonococcal survival, we incubated N927 for 2 h with sphingosine at 5 μM or DMSO as solvent control in two different media (phosphate-free Hepes and PPM+), and plated the suspension on GC agar plates for CFU determination (Figure 3B). Even sub-inhibitory concentration of sphingosine drastically reduced the survival of gonococci measured in this CFU assay.

### ω-N_3_-sphingosine Is Phosphorylated by SphK1, While 1-N_3_-sphingosine Is Not

Previous experiments demonstrated that incubation of gram-positive and gram-negative bacteria with sphingoid bases results in their association with bacterial membranes (Fischer et al., 2012; Becam et al., 2017). However, evidence for an association of sphingosine with intracellular bacteria is missing, probably due to difficulties to detect them at the single bacteria level inside the cell. We decided to use click chemistry to visualize the uptake of sphingosine by intracellular gonococci. For this purpose, new clickable sphingosine-analogs (ω-N_3_-sphingosine and 1-N_3_-sphingosine) were developed.

**Figure 4:**
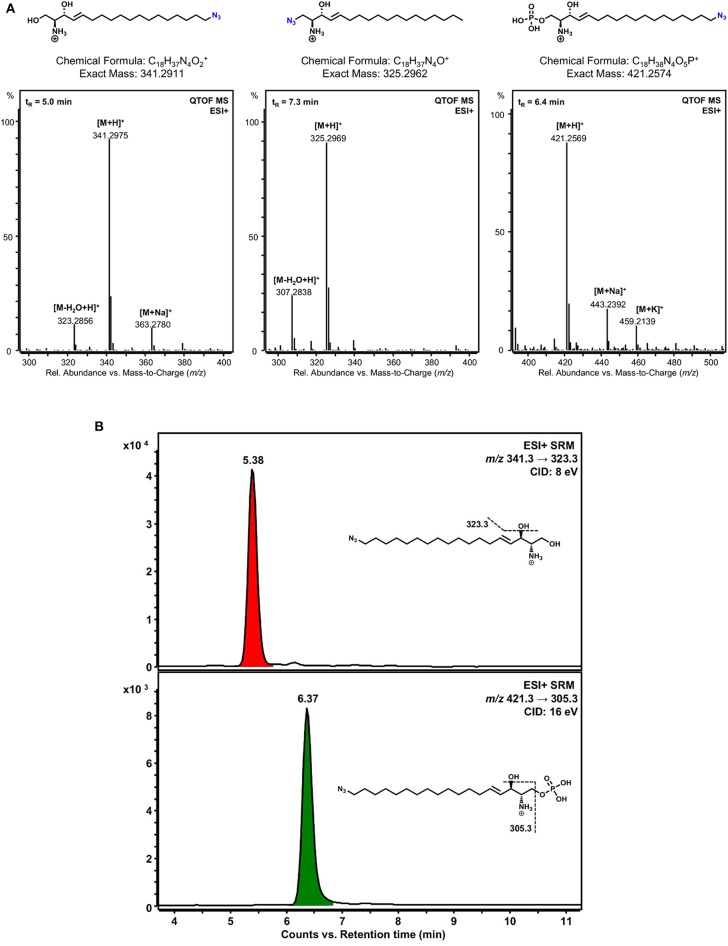
Detection of phosphorylated sphingosine analogs. **(A)** LC-HRMS verification of the identity of the clickable sphingosine analogs used and detection of a phosphorylation product of ω-N_3_-sphingosine catalyzed by SphK1 *in vitro*. Lipid extracts from incubation of 1-N_3_-sphingosine or ω-N_3_-sphingosine with SphK1 and ATP were chromatographically separated by HPLC and analyzed with a quadrupole-time-of-flight mass spectrometer (QTOF MS) operating in the positive electrospray ionization mode (ESI+). Chemical structures and mass spectra of ionized ω-N_3_-sphingosine (left), 1-N_3_-sphingosine (middle), and phosphorylated ω-N_3_-S1P (right) are shown. No phosphorylation product of 1-N_3_-sphingosine could be detected. The retention time (t_R_) of each substance analyzed is given as inset (top left) in the respective mass spectrum. **(B)** Detection of ω-N_3_-sphingosine 1-phosphate in Chang cells by LC-MS/MS. Chang cells were incubated with either 1-N_3_-sphingosine or ω-N_3_-sphingosine (10 μM) for 17 h. Lipid extracts were analyzed by selected reaction monitoring (SRM) after positive electrospray ionization (ESI+). As expected, no phosphorylation product could be detected for 1-N_3_-sphingosine (not shown). On the other hand, both ω-N_3_-sphingosine (upper chromatogram) and ω-N_3_-S1P (lower chromatogram) were present in lipid extracts of Chang cells stimulated with ω-N_3_-sphingosine. Assuming similar MS/MS responses for precursor and phosphorylation product, a conversion of about 20% occurred under the given experimental conditions. Peaks are labeled with retention time. Structural formulas and MS/MS fragmentations monitored are given as insets. CID, collision-induced dissociation.

To check the different metabolism of the two clickable sphingosine analogs, ω-N_3_-sphingosine and 1-N_3_-sphingosine, both substances were incubated separately with SphK1 and ATP. After lipid extraction, the reaction mixtures were examined by liquid chromatography high-resolution mass spectrometry (LC-HRMS). First, we have confirmed the identity of the sphingosine analogs used by means of their accurate mass-to-charge ratios (*m/z*) (Figure 4A, left and middle panel). Then the incubation approaches were analyzed for phosphorylation products. As can be seen in Figure 4A (right panel), ω-N_3_-sphingosine is phosphorylated by SphK1. The identity of the product ω-N_3_-S1P could be proven unequivocally by the protonated molecular ion [M+H]^+^ (accompanied by sodium and potassium adduct ions) in the mass spectrum with high mass accuracy (Δ*m/z* = 1.2 ppm). As expected, no corresponding product was obtained for 1-N_3_-sphingosine (not shown), because the hydroxyl group at C-1, which is targeted by SphK1, has been synthetically replaced by the azido function.

To verify that a different metabolism of the two clickable sphingosine analogs occurs not only in a cell-free assay but also *in vitro*, Chang cells were incubated with both azido-derivatives. In accordance with the cell-free kinase assay, a pronounced metabolization to the phosphorylated derivative was only detected with the ω-N_3_-sphingosine analog (Figure 4B).

**Figure 5:**
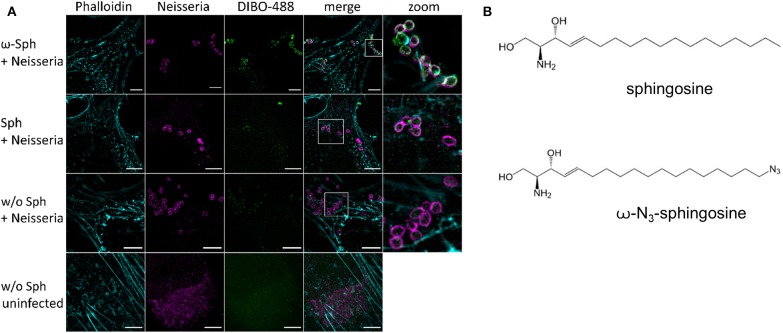
Incorporation and equal distribution of ω-azido-sphingosine into neisserial membrane of N927. **(A)** Chang cells were fed either with clickable sphingosine-analog (10 μM ω-N_3_-sphingosine, ω-Sph) or sphingosine (10 μM, Sph). These cells were infected for 4 h and then the clicking reaction with dye DIBO-488 was performed. Cells were stained for actin (Phalloidin) and immunolabeled for *Neisseria*. **(B)** Chemical structures of native sphingosine and the clickable derivate ω-N_3_-sphingosine. Images represent a minimum of eight fields viewed per replicate of three independent experiments. Bars 5 μm.

**Figure 6:**
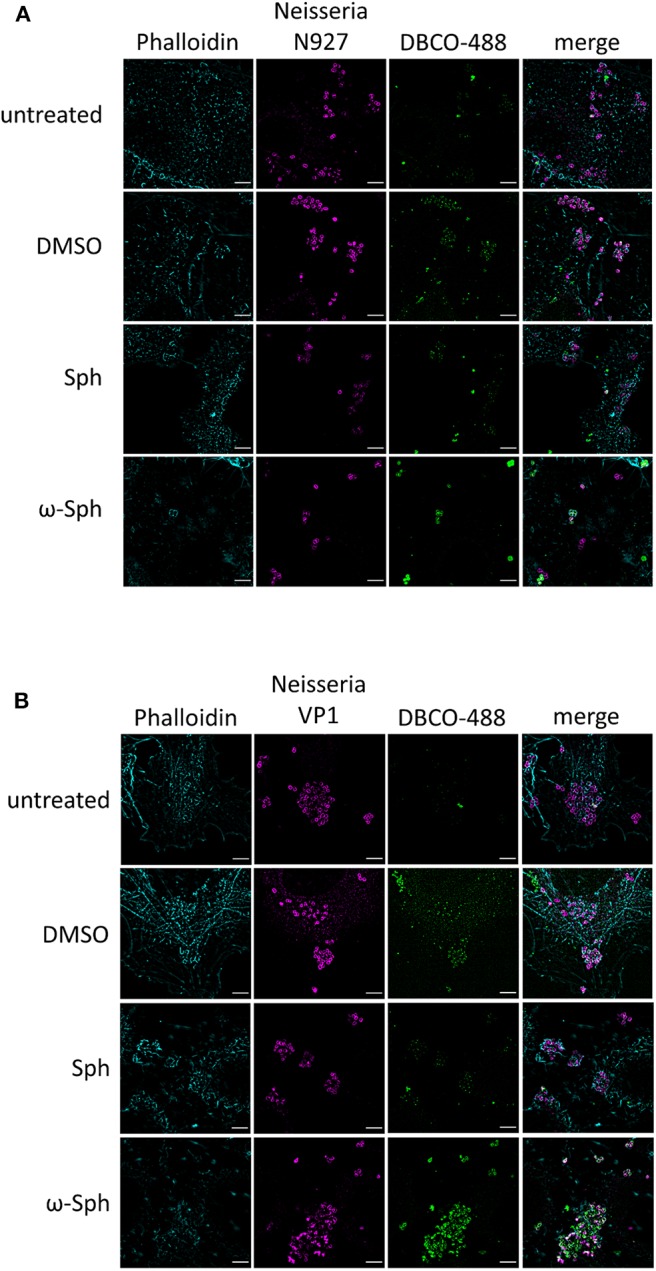
Incorporation of ω-azido-sphingosine in different infection models. Chang and End1 cells were fed either with clickable sphingosine-analog (10 μM ω-azido-sphingosine, ω-Sph), sphingosine (10 μM, Sph) or DMSO as solvent control. Cell were then infected for 4 h and the click reaction with dye DBCO-488 was performed. Cells were stained for actin cytoskeleton (Phalloidin) and *Neisseria* were immunolabeled. **(A)** Infection of End1 cells with the strain N927. **(B)** Infection of Chang cells with the clinical strain VP1. Bars 5 μm.

Since the mass spectrometry proved the functionality of the sphingosine analogs, we tested if ω-N_3_-sphingosine (ω-Sph, Figure 5B) can be taken up by intracellular bacteria (Figure 5A, Supplementary Figure 9). To test this, Chang cells were fed with ω-N_3_-sphingosine and infected with the strain N927 to check for incorporation into bacterial membranes as a possible cause for the cytotoxicity (Fischer et al., 2012). These data clearly demonstrate that the ω-N_3_-sphingosine can reach the gonococci in infected cells. Further, *Neisseria* was able to take up the analog efficiently from the host cell (Supplementary Figure 10) and incorporate it specifically into its membrane (Figure 5A). This uptake was neither restricted to the infection model nor was it strain specific since ω-N_3_-sphingosine localized to N927 in End1 cells (Figure 6A) and to VP1 in Chang cells (Figure 6B). With this experiment we can show that the biorthogonal click-reaction of the ω-N_3_-sphingosine with the alkyne dye is specific since only the combination of these two (upper panel Figure 5A, lower panel Figures 6A,B) shows a strong fluorescence of the used dye.

**Figure 7:**
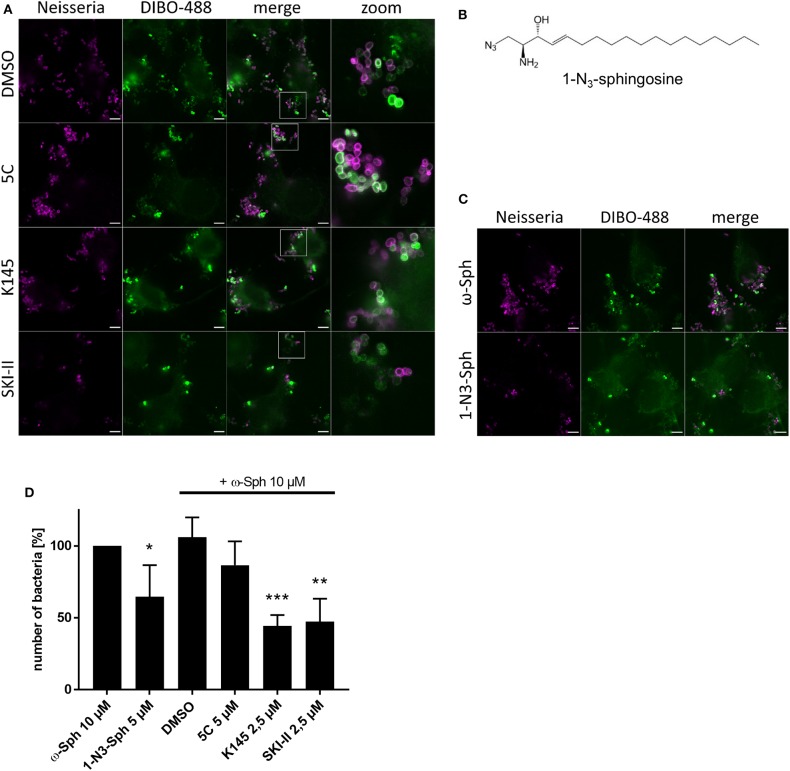
Effect of increased sphingosine concentrations on gonococci (N927) either by inhibition of SphKs **(A)** or using a sphingosine analog with the azide modification at the phosphorylation position for S1P **(B)**. **(A)** Chang cells were pre-treated with SphK inhibitors before addition of ω-N_3_-sphingosine (ω-Sph). These cells were the infected for 4 h and click reaction with DIBO-488 was performed. Gonococci were immunolabeled with anti-gonococcal antibody. **(B)** Chemical structure of the clickable derivate 1-N_3_-sphingosine (1-N_3_-Sph). **(C)** Shown are immunofluorescence images of cells treated with ω-Sph or 1-N_3_-Sph after 4 h of infection. **(D)** Quantification of gonococcal survival after 4 h of infection. To inhibit the phosphorylation of sphingosine, cells were either pre-treated with SphK inhibitors or fed with 1-N_3_-Sph. A minimum of eight fields viewed per replicate of three independent experiments were counted. Error bars show the mean ± SD of three independent experiments. *p < 0.05, **p < 0.001, ***p < 0.0001 student's *t*-test relative to ω-Sph. Images represent a minimum of eight fields viewed per replicate of three independent experiments. Bars 5 μm.

Because a greater impact on neisserial survival (Figure 1) was detected through inhibition of the conversion of sphingosine to S1P, we took the same set of inhibitors and repeated the click chemistry experiment with supplementing ω-N_3_-sphingosine (Figure 7A). In comparison to the solvent control, a general decrease in the number of gonococci was seen for the inhibitors K145 and SKI-II. Moreover, we repeatedly observed a lower intensity of neisserial antibody-staining for these inhibitors compared to solvent control or the treatment with 5C. To be sure that the observed effect is due to sphingosine, a second clickable analog, 1-N_3_-sphingosine (1-N_3_-Sph) was used. The azide-modification of this analog is introduced on the head group of the sphingosine backbone by replacing the hydroxyl group, which normally is phosphorylated by SphKs (Figure 7B). This specific acidification leads to an indirect inhibition of the metabolization of sphingosine to S1P (Figure 4A). 1-N_3_-sphingosine was therefore expected to have a stronger toxic effect on *Neisseria* compared to ω-azido-functionalized sphingosine (see Supplementary Figure 11A). Chang cells were fed with the subtoxic concentration of 5 μM 1-N_3_-sphingosine (Supplementary Figure 11). The comparison of both sphingosine-analogs after 4 h of infection shows a drastic reduction of gonococci, which have incorporated 1-N_3_-sphingosine into their membrane (Figures 7C,D). Taken together, these data show that invasive *N. gonorrhoeae* take-up host-cell sphingosine, which is incorporated into bacterial membranes and reduces survival of gonococci.

## Discussion

The role of SMases during host cell invasion of *N. gonorrhoeae* has been firmly established for PorB_IA_-dependent invasion in Chang cells and fibroblast (Faulstich et al., 2015) and for Opa-dependent invasion in RT-112 human bladder tumor cells (Grassmé et al., 1997). Here, we investigated the role of ceramide downstream signaling pathways and found that the disruption by inhibiting sphingosine kinases strongly affected the survival of intracellular gonococci. This suggested that host-cell sphingosine might exert an inhibitory effect on invasive *N. gonorrhoeae*. A bactericidal effect of sphingosine on different pathogenic bacteria has previously been demonstrated (Drake et al., 2008; Rollin-Pinheiro et al., 2016; Becam et al., 2017). However, all these experimental data were obtained by incubating bacteria with different concentrations of sphingosine in culture media. It was therefore not clear whether sphingosine directly interacts with bacteria inside their host cells. Here we show using click chemistry with novel sphingosine analogs, that sphingosine co-localizes with the surface of intracellular bacteria, demonstrating that host cell sphingosine can reach intracellular bacteria where it may exert its bactericidal effect. Since SMases play a role in Opa-dependent and—independent gonococcal invasion pathways, our findings may generally be relevant for the intracellular survival of those gonococci that activate SMase pathways during invasion.

To investigate the role of sphingosine in infection, we interfered with the metabolization of sphingosine to sphingosine-1-phosphate (S1P) by inhibiting the sphingosine metabolizing sphingosine kinases to increase the intracellular levels of sphingosine. The clear effect on the intracellular survival of gonococci indicated that an imbalance in ceramide metabolism affected either the host cells or the bacterial physiology. To discriminate between these two possibilities, we aimed to demonstrate a direct interaction of sphingosine with gonococci. This could only be achieved by developing novel clickable sphingosine analogs that allowed the highly specific detection of sphingosine in the infected cell. These analyses revealed the incorporation of sphingosine equally distributed at the surface of gonococci, presumably by incorporation into the bacterial membrane. We speculate that the integration of sphingosine into the bacterial membranes interferes with intracellular survival of the bacteria, probably by immediate membrane damage and subsequent lysis of the bacteria. This is in line with the observation, that the binding efficiency of the neisserial antibody used to detect gonococci was strongly reduced if the bacteria stained positive for sphingosine, in line with the previously postulated occurrence of “microlesions” in bacterial membranes upon the incorporation of sphingosine (Fischer et al., 2012). However, the exact bacteria killing mechanism of sphingosine has not been elucidated yet.

The inhibitor studies showed that the intracellular survival of gonococci is dependent on the activity of SphKs, particularly on SphK2. This is in line with less intracellular survival of gonococci upon treatment with the K145 and SKI-II inhibitors in gentamicin protection assays. Although both SphKs catalyze the conversion of sphingosine, they have different kinetic properties and expression patterns suggesting distinct physiological functions (Spiegel and Milstien, 2007). These differences have a strong influence on the intracellular concentrations of sphingosine as we could demonstrate by mass spectrometric analysis (Supplementary Figure 6). These differences appear also to affect gonococcal survival, since the inhibition of SphK1 caused no change in sphingosine levels (Supplementary Figure 6) and induced no survival defect in gonococci (Figure 1; Supplementary Figure 1). It is possible that gonococci activate SphK2 to protect their intracellular niche against the harmful effect of sphingosine. However, inhibitor studies alone are certainly not sufficient to predict mechanisms. All our attempts to generate SphK negative Chang cells failed so far due to compensatory upregulation of SphK1 in SphK2 knockdowns and vice versa, or the failure to efficiently silence both kinases simultaneously (not shown). We therefore made use of the modified sphingosine analog, which carries the azido group instead of the hydroxyl group in position 1 (1-N_3_-sphingosine) and can therefore not be converted to S1P by sphingosine kinases. Addition of this derivative had a significantly stronger bactericidal effect than the ω-N_3_-sphingosine that can be metabolized by SphK (Figure 7) as the unmodified sphingosine. We therefore argue that both, the inhibitor experiments and the non-phosphorylatable sphingosine derivatives support a role of sphingosine in the inhibition of the survival of intracellular gonococci.

Although direct evidence is missing so far, it is tempting to speculate that sphingolipid derivatives may play a more prominent and direct role in the control of intracellular bacteria than previously anticipated. Some bacteria have been demonstrated to incorporate certain types of sphingolipids into their membranes as a lipid source and essential constituent. Examples are obligate intracellular *Chlamydia*, which use host cells ceramides to build their own membranes and the bacteria containing vacuoles (inclusions) (Derré et al., 2011; Banhart et al., 2019). Interference with sphingolipid transport to these bacteria strongly interferes with their normal development (Koch-Edelmann et al., 2017). In contrast, our data suggest that sphingolipids can be part of the host cells defense against intracellular bacteria. The clickable sphingosine probes described here are useful tools to explore the potential role of sphingosine in the host cell defense against other intracellular bacteria.

## Materials and Methods

### Mammalian Cell Lines

The human epithelial conjunctival cell line (Chang) was used as neisserial infection model. Chang cells were cultured in RPMI-1640 medium (Gibco Life Technologies, Karlsruhe, Germany) supplemented with 10% fetal calf serum (FCS, Gibco Life Technologies) at 37°C and 5% CO_2_ in a humidified atmosphere. For validation, the gentamicin protection assay, the infectivity assay and click chemistry visualization were repeated with the immortalized human endocervical cell line End1/E6E7, which was cultured as described (Fichorova et al., 1997). Briefly, these cells were cultured in DMEM/F12 (Gibco Life Technologies, Karlsruhe, Germany) supplemented with 10% fetal calf serum (FCS, Gibco Life Technologies) in a humid atmosphere at 37° C and 5% CO_2_. All cells were grown in T75 flasks (Corning, New York, USA).

### Neisserial Strains, Culture Conditions, and Growth Curves

*N. gonorrhoeae* MS11 N927 (PorB_IA_, pili^−^, Opa^−^), FA1090 (PorB_IB_, pili^−^, Opa^−^) and clinical isolates from patients with DGI, including strain 24871 (PorB_IA_, pili^−^, Opa^−^), VP1 (PorB_IA_, pili^−^, Opa^−^) were cultivated on gonococci (GC) agar (ThermoScientific, Waltham, USA) plates supplemented with 1% vitamin mix at 37°C and 5% CO_2_ for 16 h. Liquid culture for infection was performed in proteose-peptone medium (PPM) supplemented with 1% vitamin mix and 0.5% sodium bicarbonate 8.4% solution (PPM+) at 37°C and 120 rpm. Before infecting cells, the medium of the liquid culture was changed to phosphate-free Hepes medium (Kühlewein et al., 2006) by centrifugation with 4,000 rpm for 5 min. Growth curve experiments were carried out by inoculating a liquid culture with *Neisseria* from a GC agar plate, starting at OD_550nm_ 0.15. The bacteria were grown until they reached OD_550nm_ 0.5–0.6. Cultures were then diluted to OD_550nm_ 0.1 equivalent to 0.4 × 10E7 bacteria per mL and supplemented with inhibitors, sphingosine or clickable sphingosine analogs at different concentrations and DMSO as solvent control. Additionally, a positive control with 15 μg/ml kanamycin was included. Bacterial growth was measured every hour.

### Infection Assays

Cells were seeded into 24-well plates and grown to 70% confluence. On the day of infection, the medium wash changed to Hepes medium to provide phosphate-free conditions and PorB_IA_-dependent invasion. The cells were incubated for about 20 min before inhibitor treatment of 2 h. The used inhibitors were 5C (Santa Cruz Biotechnology, Santa Cruz, USA), K145 (Sigma-Aldrich, St. Louis, USA), and SKI-II (abcam, Cambridge, United Kingdom). All inhibitors were resolved in dimethyl sulfoxide (DMSO). Infections were performed at an MOI of 50 for 1 h at 37°C and 5% CO_2_ in the presence of solvent or inhibitor. The infection was stopped by washing the cells three times with Hepes medium, followed by gentamicin protection assay as previously described (Kühlewein et al., 2006). Briefly, to assess total quantity of adherent and invasive bacteria cells were lysed with saponin for about 7 min. For Chang and End1/E6E7 cells 1 and 1.5% saponin were used, respectively. The bacteria suspensions were diluted and plated on GC agar plates. Colony forming units (CFUs) were counted 24 h after plating. To quantify exclusively intracellular *Neisseria*, cells were incubated with 150 μg/ml gentamicin for 2 h, followed by saponin lysis as described earlier.

### Differential Immunofluorescence Staining

To discriminate between intracellular and extracellular bacteria, differential immunofluorescence staining was performed and analyzed manually or by using an Operetta High-Content Imaging System (PerkinElmer, Waltham, USA) for automated counting. Cells were seeded either on 12 mm cover slips in 12-wells (Corning, New York, USA) or in μ-Plate 24 Well Black plates (ibidi, Martinsried, Germany), respectively, and grown to 70–80% confluence. The staining procedure was the same for both methods. The cells were treated and infected with *Neisseria* at MOI 10 as previously described (see Infection assays). Infection was stopped by washing with PBS. Then the samples were fixed with 4% PFA for 15 min in the dark at room temperature, followed by washing three times with PBS and subsequently blocked with 1% BSA in PBS for 45 min at room temperature. All following antibody staining steps were performed at room temperature and in the dark. After blocking, the cells were incubated in a 1:200 dilution of the primary antibody, polyclonal rabbit anti-gonococcal (US biological, Salem, USA) in 1% bovine serum albumin in PBS, for 1 h to detect the extracellular bacteria. The cells were washed twice with PBS and once with 1% BSA in PBS before they were incubated with the Cy5 conjugated secondary anti-rabbit antibody (1:100 in 1% BSA in PBS, dianova, Hamburg, Germany) for 1 h. Afterwards the cells were washed again and permeabilized with 0.1% Triton-X in PBS for 15 min. After another washing step, the cells were blocked in 1% BSA in PBS for 45 min. Next, the extra- and intracellular bacteria were immunolabeled using the anti-gonococcal primary antibody (1:200 in 1% BSA in PBS) followed by washing steps. Together with the Cy2-conjugated secondary anti-rabbit antibody (1:100 in 1% BSA in PBS, dianova, Hamburg, Germany), the actin cytoskeleton (Alexa Fluor® 555 Phalloidin, 1:100 in 1% BSA in PBS, ThermoScientific, Waltham, USA) and the DNA (Dapi, 1:3,000 1% BSA in PBS, ThermoScientific, Waltham, USA) were stained for 45 min. The wells were washed again and for manually counting the cover slips were embedded in mowiol on object slides, dried overnight and analyzed by confocal microscopy (63× oil immersion objective) with a Leica TCS SPE. The Operetta samples were stored in 4% PFA at 4°C until they were analyzed.

### Apoptosis Analysis and Flow Cytometry

Chang cells (1 × 10^5^) were seeded in 12-wells and grown to 80–90% confluence. The medium was replaced by phosphate-free Hepes medium before cells were treated with inhibitors or analogs. For the inhibitor experiments, cells were treated with the indicated concentrations of inhibitors or DMSO as control for 2 h. The click chemistry experiments were carried out by feeding the cells the indicated concentrations of the sphingosine analogs for 30 min. Then the medium was exchanged for Hepes medium for 1, 2, or 4 h according to infection times. Subsequently the cells were trypsinized and collected. After a centrifugation step (800 g, 5 min at 4°C) the cell pellet was resuspended in 500 μl PBS and 1 μl propidium iodide (PI; 250 μg/ml, ICT, Bloomington, USA) was added. The cells were incubated for 10 min in the dark. Afterwards the cells were put on ice and one sample was treated with 0.1% Triton-X as a negative control shortly before analysis. The analysis was performed using FACS AriaIII (BD, New Jersey, USA).

### Click Chemistry Reaction and Visualization

The visualization was performed by using biorthogonal copper-free click chemistry, where unnatural azide groups on the molecules of interest are conjugated to alkyne dyes.

Cells were seeded on 15 mm^2^ cover slips in 12-wells (Corning, New York, USA) and grown to a confluency of about 70%. The media was changed to Hepes medium and the cells were fed with functionalized sphingosines (ω-N_3_-sphingosine or 1-N_3_-sphingosine) or D-sphingosine (Santa Cruz, Dallas, USA) for 30 min at 37 °C and 5% CO_2_. D-sphingosine and ω-N_3_-sphingosine (Lang et al., 2020) were applied with a final concentration of 10 μM each. Because of its higher toxicity, 1-N_3_-sphingosine was used at 5 μM. The sphingosine compounds were dissolved in DMSO. For the inhibitor studies, cells were treated with the inhibitors (5 μM 5C, 2.5 μM K145 and 2.5 μM SKI-II) 30 min prior to sphingosine feeding. After the indicated feeding time, cells were washed briefly with warm Hepes medium to remove residual analog from the media. Inhibitors were applied again at the indicated concentrations. The infection was performed with an MOI of 30 for 4 h. To stop the infection after the indicated infection time, cells were washed three times with Hepes medium and the copper-free clicking reaction with 5 μM Click-IT Alexa Fluor® 488 DIBO alkyne dye (ThermoScientific, Waltham, USA) or 5 μM Alexa Fluor® 488-DBCO (Jena Bioscience, Jena, Germany) was performed for 30 min in a humidified atmosphere at 37°C. The cells were washed two times with PBS and fixed with 4% PFA for 30 min. Afterwards the cells were washed again with PBS and permeabilized with 0.1% Triton-X in PBS for 15 min. After another washing step, the cells were blocked in 1% BSA in PBS for 1 h. The gonococci were immunolabeled using polyclonal rabbit anti-gonococcal primary antibody (1:100 in 1% BSA in PBS) for 1 h followed by brief washing with PBS. Together the Atto647 anti-rabbit antibody (1:300 in 1% BSA in PBS, Sigma-Aldrich, St. Louis, USA), the actin cytoskeleton (Alexa Fluor® 555 Phalloidin, 1:100 in 1% BSA in PBS, ThermoScientific, Waltham, USA) was stained for 1 h. The staining was completed by post-fixation with 4% PFA for 30 min. The samples were washed again and the cover slips were embedded in mowiol on object slides and dried overnight. Images were acquired on a Zeiss (Oberkochen, Germany) ELYRA S.1 SR-SIM structured illumination platform using a Plan-Apochromat 63× oil-immersion objective with a numerical aperture of 1.4. Reconstruction of superresolution images were performed using the ZEN image-processing platform with a SIM module.

To quantify the effect of 1-N_3_-sphingosine and the combination of SphK-inhibitors with ω-N_3_-sphingosine on the survival of *Neisseria*, superresolution images of three independent experiments were taken and the signal of the anti-*Neisseria* antibody were counted blinded to calculate the number of gonococci per cell. The cell number of each image was assessed by cytoskeleton staining and the clicked sphingosine signal. For each treatment of the biological replicates a minimum number of eight microscopy images were analyzed.

### *In vitro* Phosphorylation Assay and Mass Spectrometry

To investigate the enzyme-driven phosphorylation of 1-N_3_-sphingosine and ω-N_3_-sphingosine, we incubated both functionalized sphingosine analogs (10 μM each) separately with 4 mM ATP (Sigma-Aldrich, Taufkirchen, Germany) and 10 U recombinant, human SphK1 (Sigma-Aldrich) in 40 mM HEPES-NaOH buffer (pH 7.4) for 1 h at 37°C under gentle shaking. Afterwards lipids were extracted and resulting samples were subjected to LC-HRMS analysis as described recently (Wigger et al., 2019). Briefly, chromatographic separations were performed with an Agilent 1260 Infinity HPLC coupled to an Agilent 6530 quadrupole-time-of-flight mass spectrometer (QTOF MS) operating in the positive electrospray ionization (ESI+) mode (Agilent Technologies, Waldbronn, Germany). The QTOF MS was operated in full scan mode, acquiring data in the *m/z* range of 100–750 with a scan rate of 2 spectra/s.

### SphK Inhibition Assay and Phosphorylation Capability for ω-N_3_-Sph in Chang Cells

Chang cells were cultured in RPMI-1640 medium (Merck Millipore, Darmstadt, Germany) supplemented with 10% FBS (PAN-Biotech, Aidenbach, Germany) and 1% penicillin-streptomycin (Sigma-Aldrich, Taufkirchen, Germany) at 37°C and 5% CO_2_ in a humidified atmosphere. The day before the experiment, 2 × 10^6^ cells were seeded in 10 cm cell culture dishes. After reaching 70–80% confluence, cells were incubated for 1 h with the SphK inhibitors 5C, K145 or SKI-II (all from Sigma-Aldrich) in two concentrations (2.5 and 5 μM) in triplicates. DMSO served as solvent control. An additional subset of triplicates was treated either with 1-N_3_-Sph or ω-N_3_-Sph (final concentration: 10 μM). Pre-stimulated cells were then incubated with 100 μM palmitic acid (16,16,16-d_3_) from Cortecnet (Voisins-le-Bretonneux, France) applied as a BSA complex. For cells treated with azido-sphingosine analogs the addition of labeled palmitate was omitted. After 16 h of incubation at 37°C, media were removed, cells were washed with 5 ml PBS and subsequently harvested in 500 μl MeOH. Sphingolipids were extracted using 1.5 ml methanol/chloroform (2:1,v:v) as described (Gulbins et al., 2018). The extraction solvent contained sphingosine-d_7_ (Sph-d_7_) and C16-d_31_-sphingomyelin (C16-d_31_ SM) (both Avanti Polar Lipids, Alabaster, USA) as internal standards. LC-ESI(+)-MS/MS analyses were conducted with a 1260 Infinity HPLC coupled to a 6490 triple-quadrupole mass spectrometer (both Agilent Technologies, Waldbronn, Germany) as recently described (Naser et al., 2020). The following mass transitions were recorded (collision energies in parentheses): *m/z* 303.3 → 285.3 for Sph-d_3_ (8 eV), *m/z* 305.3 → 287.3 for dhSph-d_3_ (12 eV), *m/z* 307.3 → 289.3 for Sph-d_7_ (8 eV), *m/z* 325.3 → 307.3 for 1-N_3_-Sph (8 eV), *m/z* 341.3 → 323.3 for ω-N_3_-Sph (8 eV), *m/z* 405.3 → 264.3 for 1-N_3_-S3P (16 eV), *m/z* 421.3 → 305.3 for ω-N_3_-S1P (16 eV), *m/z* 731.6 → 184.1 for C18 SM (25 eV), and *m/z* 734.8 → 184.1 for C16-d_31_ SM (25 eV). Detected amounts of *de novo* formed deuterated long-chain bases were normalized to the C18 SM content of the lipid extract.

### Statistical Analysis

Statistical significance was calculated with unpaired Student *t* test with ^****^p < 0.0001, ^***^p < 0.001, ^**^p < 0.01, ^*^p < 0.05.

## Data Availability Statement

The datasets generated for this study are available on request to the corresponding author.

## Author Contributions

FSo performed the infection and clicking experiments. FSo, TK, and KP performed the imaging experiments. FH performed the *in vitro* sphingosine experiments. JF and PP synthesized and purified the azido-functionalized sphingosines. FSc and BK performed *in vitro* metabolization and mass spectrometry. JS and TR conceived the study and participated in all stages of the work. FSo and TR wrote the manuscript.

## Conflict of Interest

The authors declare that the research was conducted in the absence of any commercial or financial relationships that could be construed as a potential conflict of interest.
