# Supplementary material for: A Role of Sphingosine in the Intracellular Survival of Neisseria gonorrhoeae
Source: Front Cell Infect Microbiol. 2020 May 12;10:215. doi: 10.3389/fcimb.2020.00215 (PMC7235507; doi:10.3389/fcimb.2020.00215)
Supplement: Supplementary file 1 [file Presentation_1.PPTX]

## Slide 1
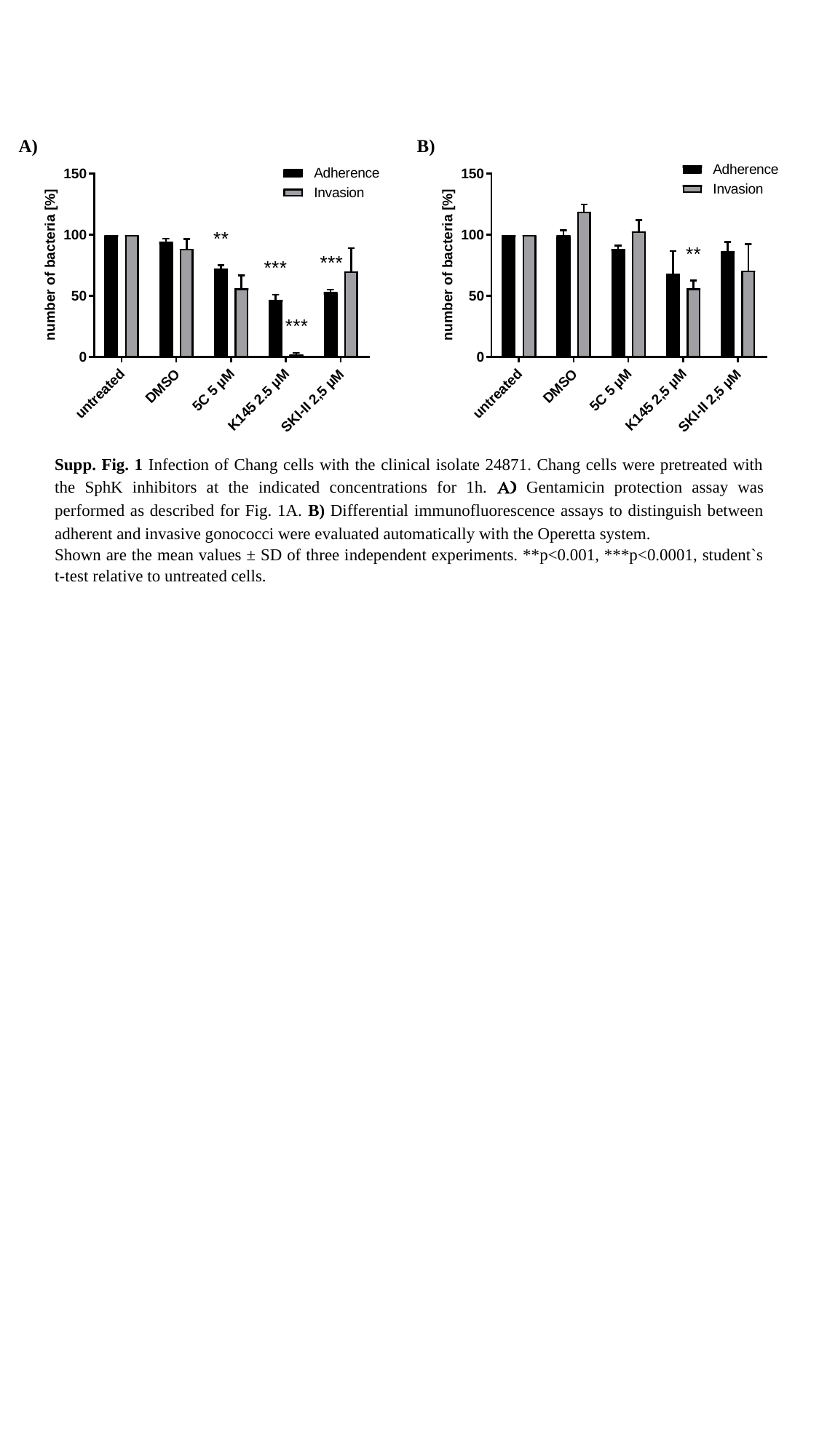

A)
B)
Supp. Fig. 1 Infection of Chang cells with the clinical isolate 24871. Chang cells were pretreated with the SphK inhibitors at the indicated concentrations for 1h. A) Gentamicin protection assay was performed as described for Fig. 1A. B) Differential immunofluorescence assays to distinguish between adherent and invasive gonococci were evaluated automatically with the Operetta system.
Shown are the mean values ± SD of three independent experiments. **p<0.001, ***p<0.0001, student`s t-test relative to untreated cells.

## Slide 2
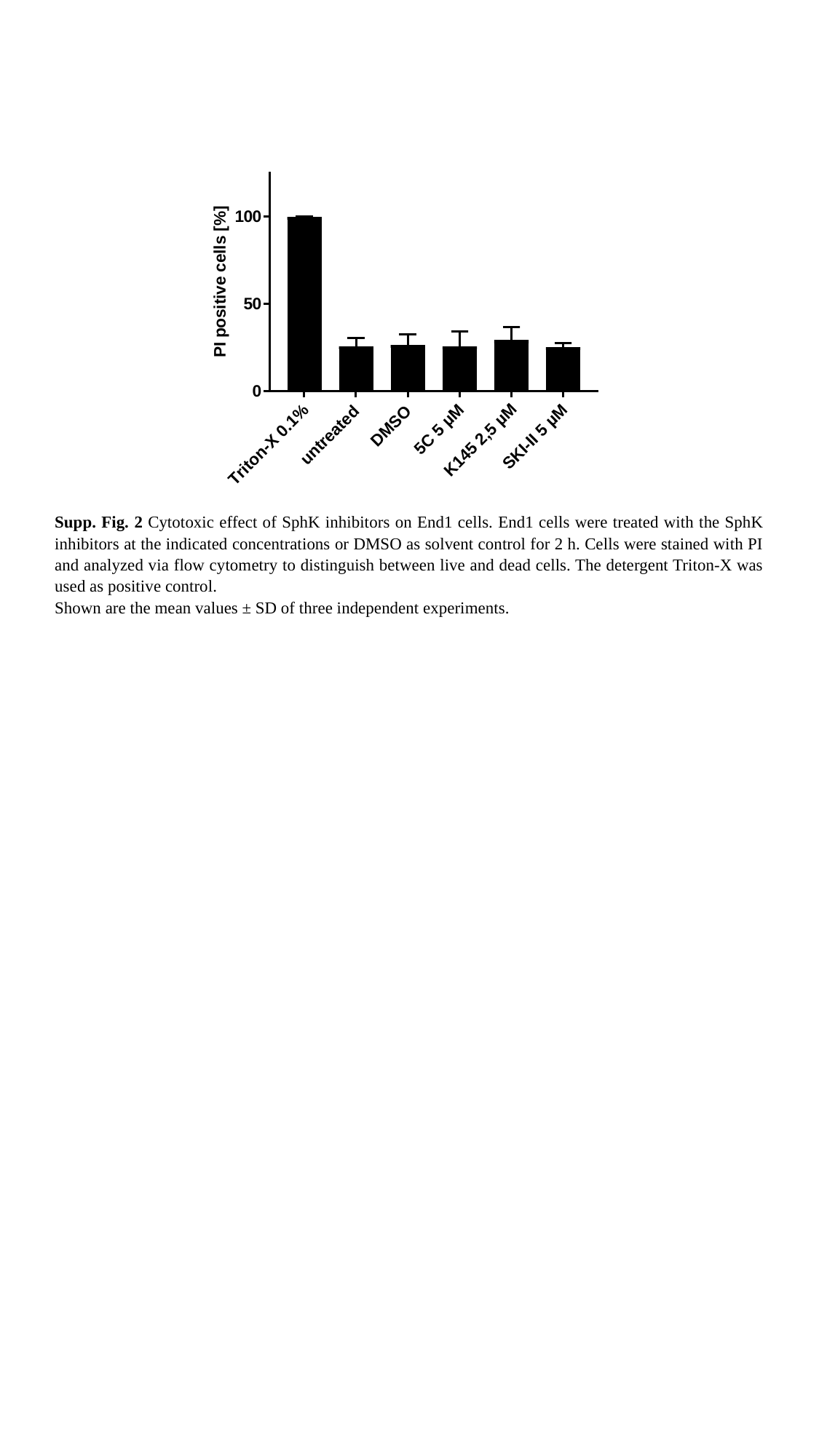

Supp. Fig. 2 Cytotoxic effect of SphK inhibitors on End1 cells. End1 cells were treated with the SphK inhibitors at the indicated concentrations or DMSO as solvent control for 2 h. Cells were stained with PI and analyzed via flow cytometry to distinguish between live and dead cells. The detergent Triton-X was used as positive control.
Shown are the mean values ± SD of three independent experiments.

## Slide 3
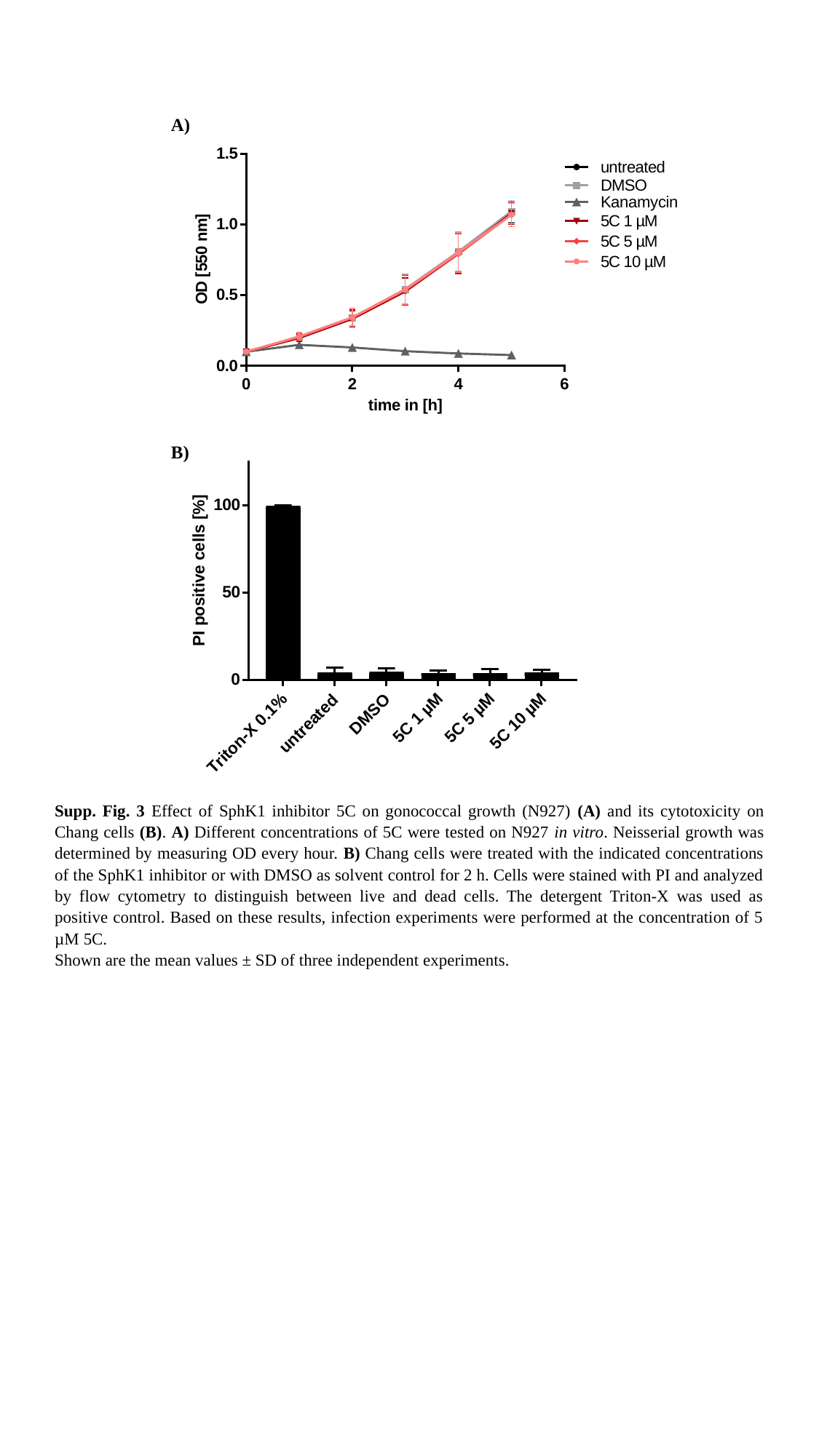

A)
B)
Supp. Fig. 3 Effect of SphK1 inhibitor 5C on gonococcal growth (N927) (A) and its cytotoxicity on Chang cells (B). A) Different concentrations of 5C were tested on N927 in vitro. Neisserial growth was determined by measuring OD every hour. B) Chang cells were treated with the indicated concentrations of the SphK1 inhibitor or with DMSO as solvent control for 2 h. Cells were stained with PI and analyzed by flow cytometry to distinguish between live and dead cells. The detergent Triton-X was used as positive control. Based on these results, infection experiments were performed at the concentration of 5 µM 5C.
Shown are the mean values ± SD of three independent experiments.

## Slide 4
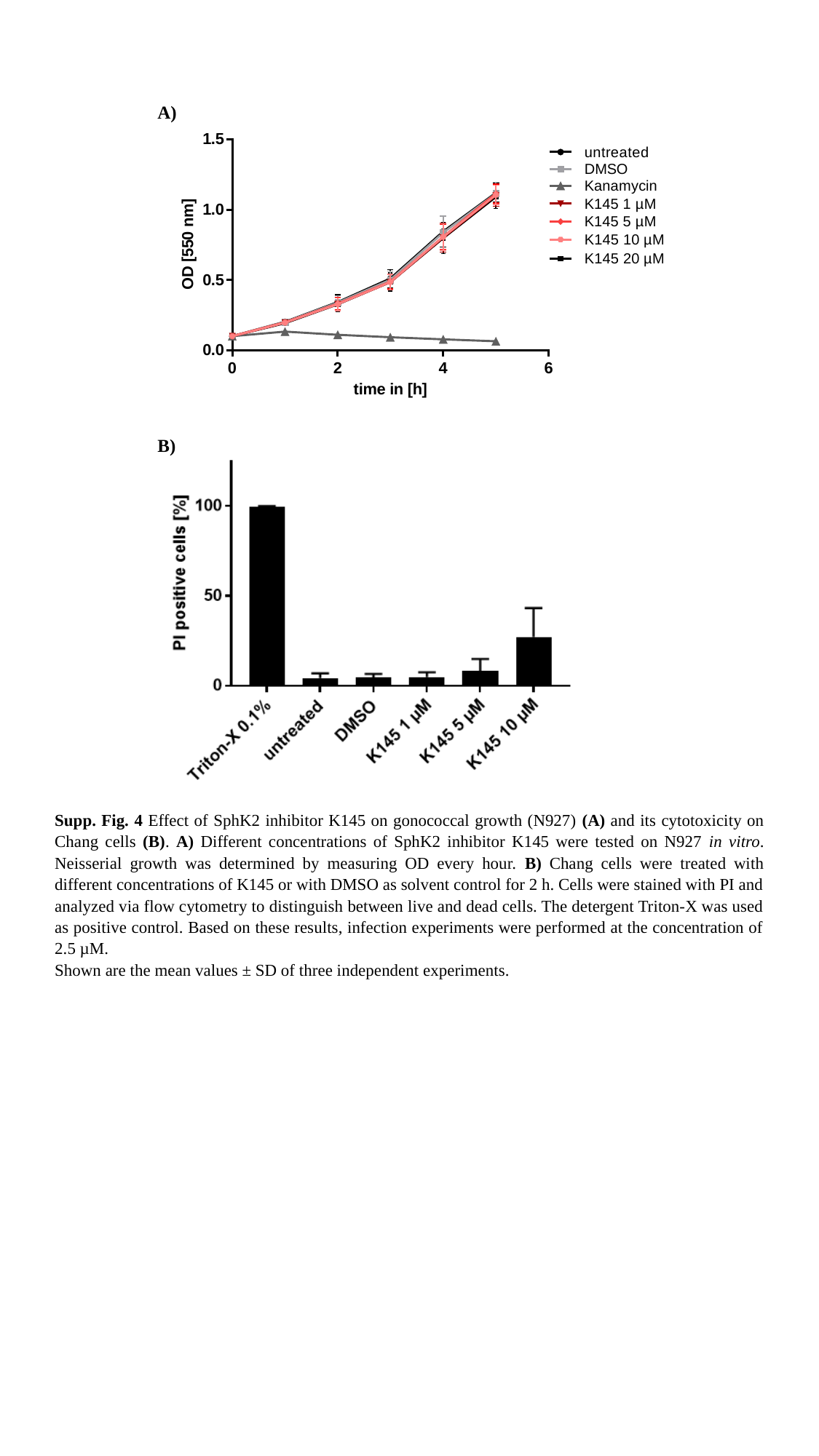

A)
B)
Supp. Fig. 4 Effect of SphK2 inhibitor K145 on gonococcal growth (N927) (A) and its cytotoxicity on Chang cells (B). A) Different concentrations of SphK2 inhibitor K145 were tested on N927 in vitro. Neisserial growth was determined by measuring OD every hour. B) Chang cells were treated with different concentrations of K145 or with DMSO as solvent control for 2 h. Cells were stained with PI and analyzed via flow cytometry to distinguish between live and dead cells. The detergent Triton-X was used as positive control. Based on these results, infection experiments were performed at the concentration of 2.5 µM.
Shown are the mean values ± SD of three independent experiments.

## Slide 5
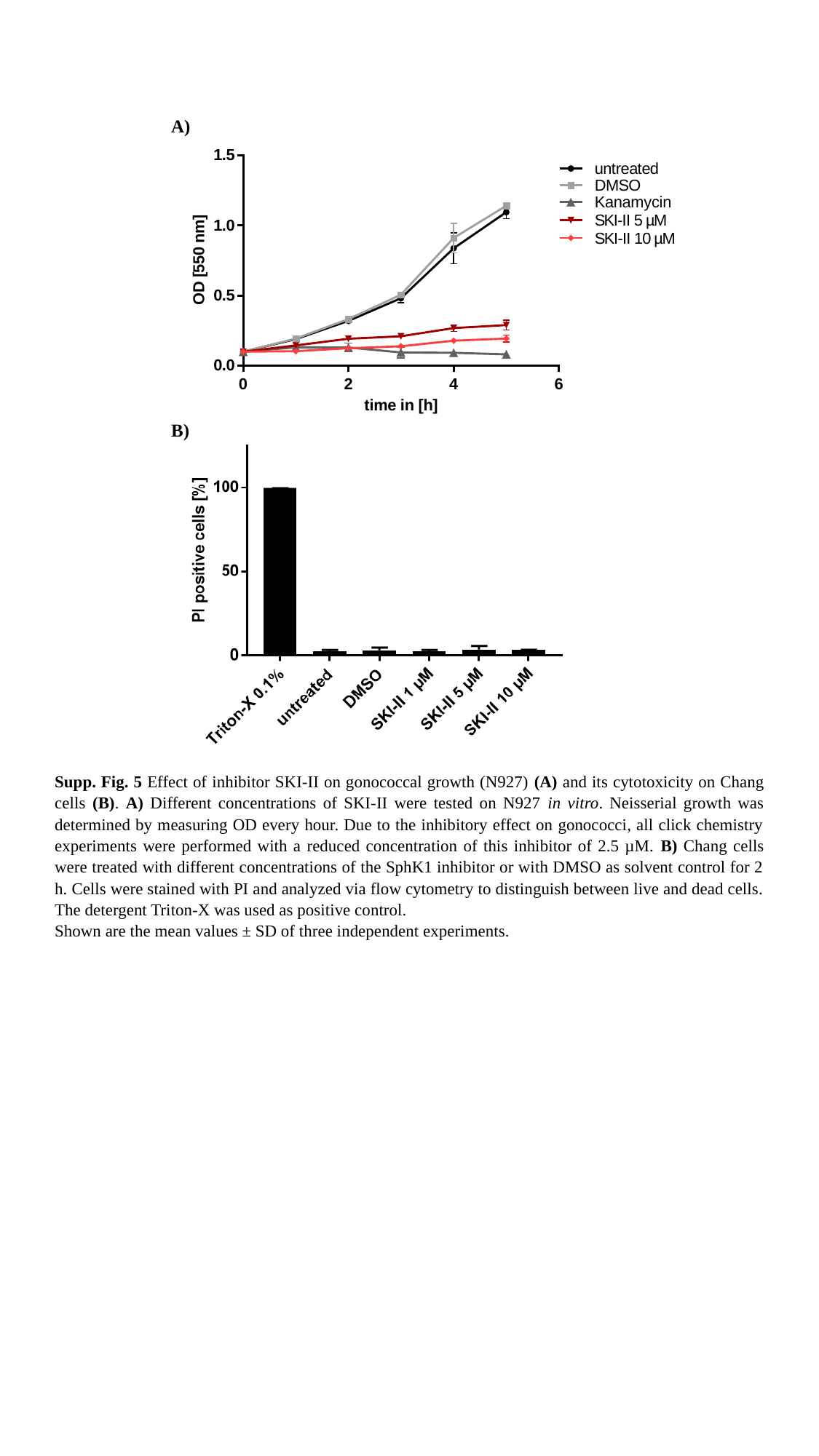

A)
B)
Supp. Fig. 5 Effect of inhibitor SKI-II on gonococcal growth (N927) (A) and its cytotoxicity on Chang cells (B). A) Different concentrations of SKI-II were tested on N927 in vitro. Neisserial growth was determined by measuring OD every hour. Due to the inhibitory effect on gonococci, all click chemistry experiments were performed with a reduced concentration of this inhibitor of 2.5 µM. B) Chang cells were treated with different concentrations of the SphK1 inhibitor or with DMSO as solvent control for 2 h. Cells were stained with PI and analyzed via flow cytometry to distinguish between live and dead cells. The detergent Triton-X was used as positive control.
Shown are the mean values ± SD of three independent experiments.

## Slide 6
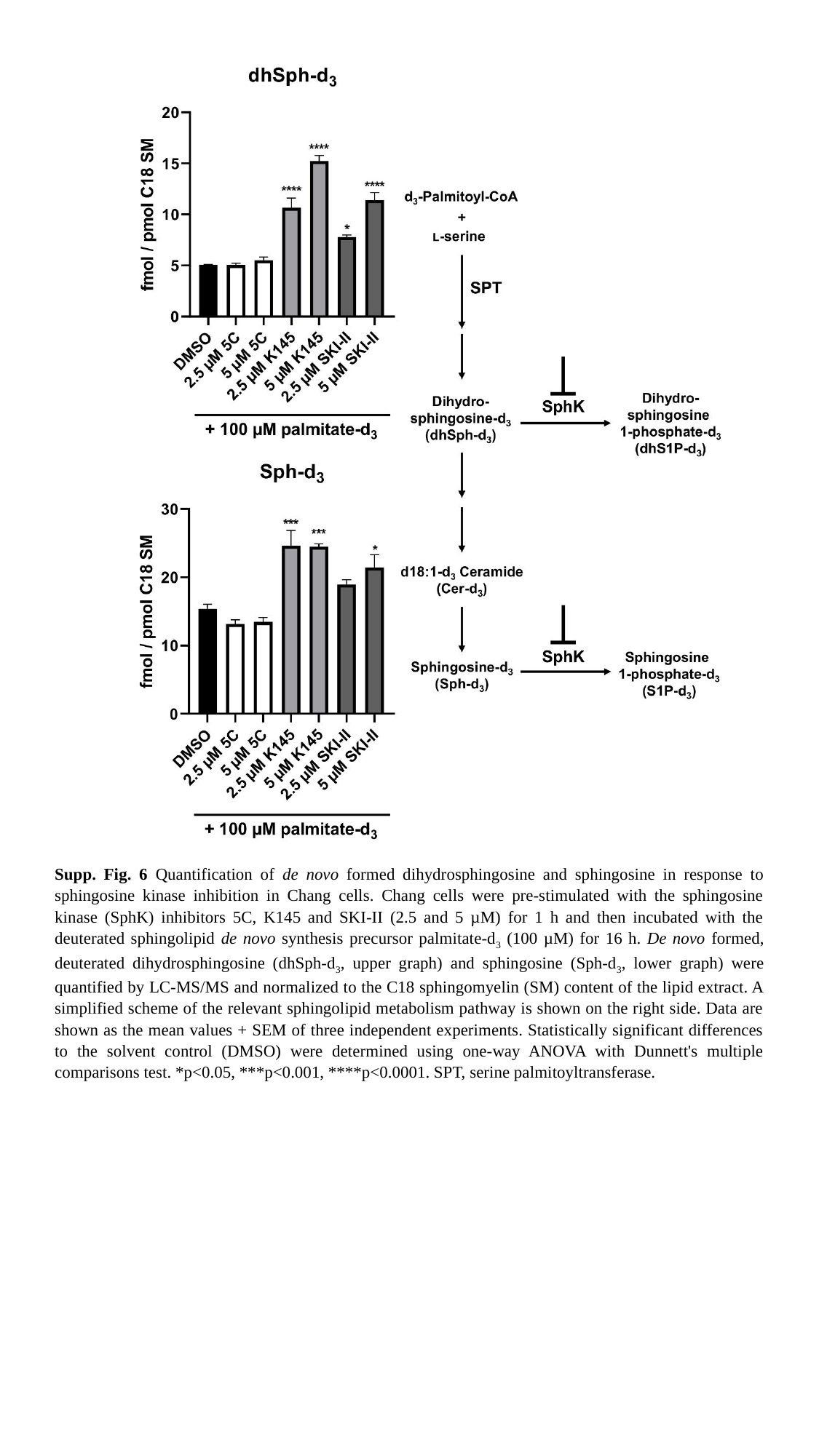

Supp. Fig. 6 Quantification of de novo formed dihydrosphingosine and sphingosine in response to sphingosine kinase inhibition in Chang cells. Chang cells were pre-stimulated with the sphingosine kinase (SphK) inhibitors 5C, K145 and SKI-II (2.5 and 5 µM) for 1 h and then incubated with the deuterated sphingolipid de novo synthesis precursor palmitate-d3 (100 µM) for 16 h. De novo formed, deuterated dihydrosphingosine (dhSph-d3, upper graph) and sphingosine (Sph-d3, lower graph) were quantified by LC-MS/MS and normalized to the C18 sphingomyelin (SM) content of the lipid extract. A simplified scheme of the relevant sphingolipid metabolism pathway is shown on the right side. Data are shown as the mean values + SEM of three independent experiments. Statistically significant differences to the solvent control (DMSO) were determined using one-way ANOVA with Dunnett's multiple comparisons test. *p<0.05, ***p<0.001, ****p<0.0001. SPT, serine palmitoyltransferase.

## Slide 7
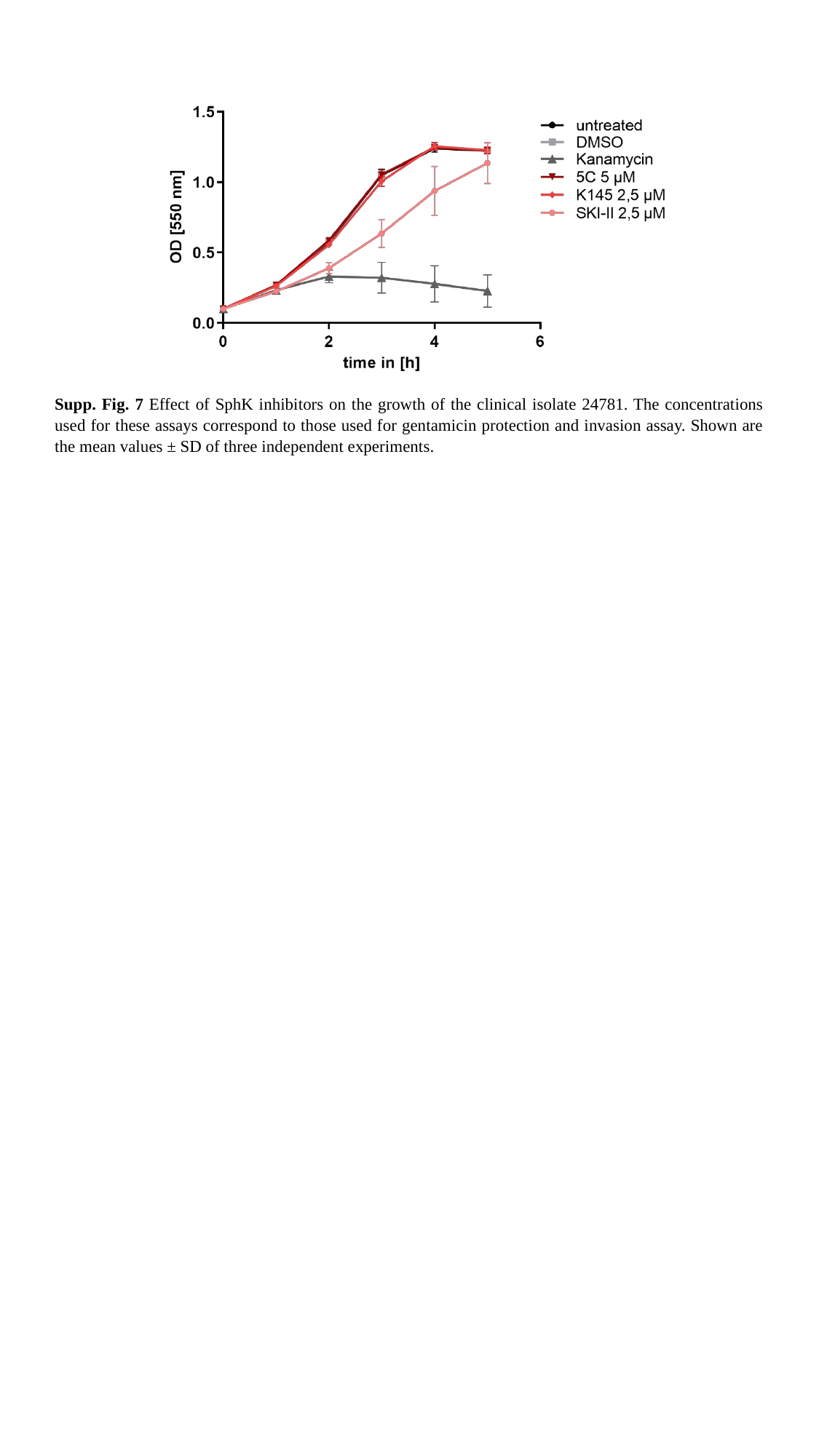

Supp. Fig. 7 Effect of SphK inhibitors on the growth of the clinical isolate 24781. The concentrations used for these assays correspond to those used for gentamicin protection and invasion assay. Shown are the mean values ± SD of three independent experiments.

## Slide 8
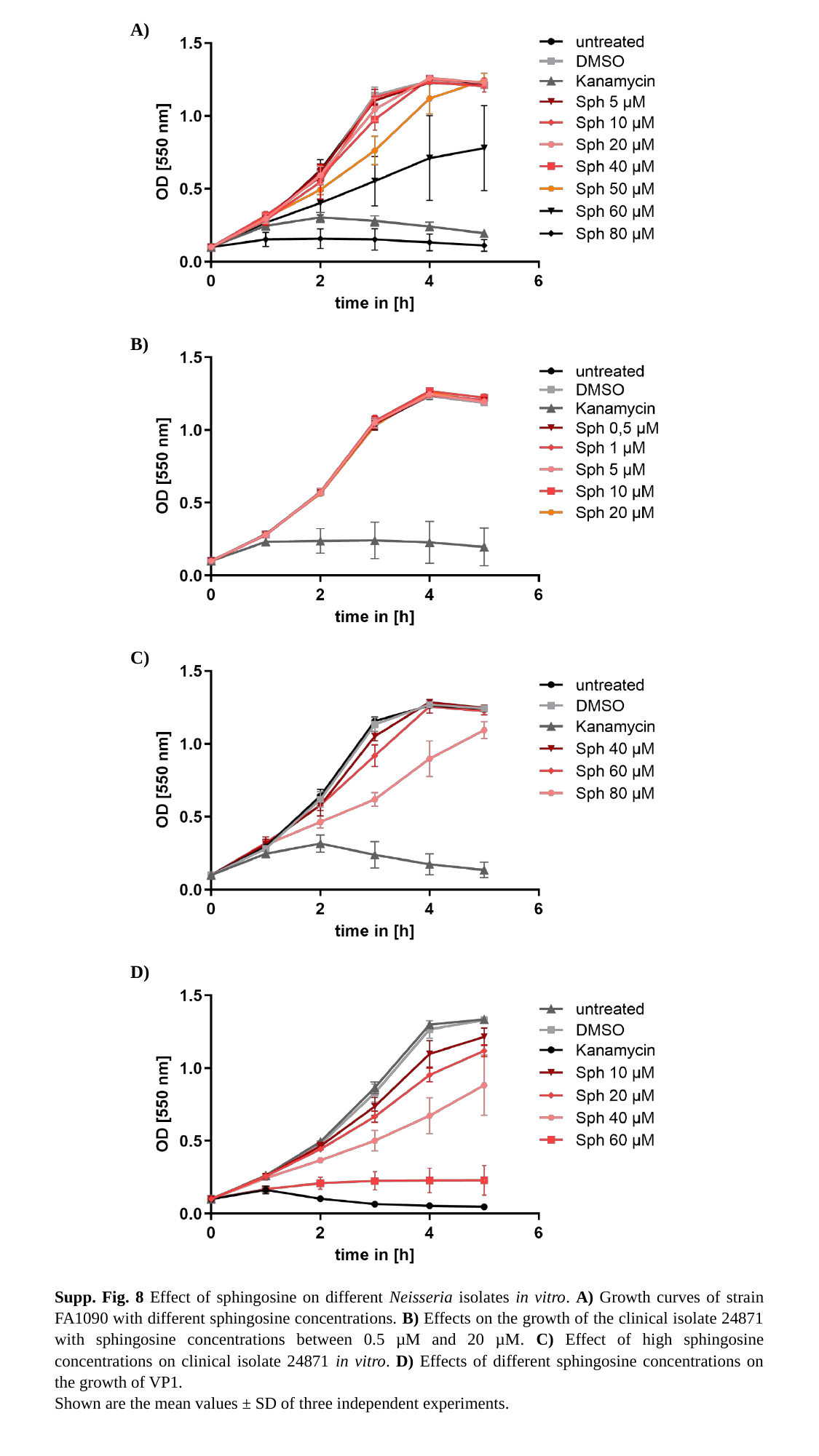

A)
B)
C)
D)
Supp. Fig. 8 Effect of sphingosine on different Neisseria isolates in vitro. A) Growth curves of strain FA1090 with different sphingosine concentrations. B) Effects on the growth of the clinical isolate 24871 with sphingosine concentrations between 0.5 µM and 20 µM. C) Effect of high sphingosine concentrations on clinical isolate 24871 in vitro. D) Effects of different sphingosine concentrations on the growth of VP1.
Shown are the mean values ± SD of three independent experiments.

## Slide 9
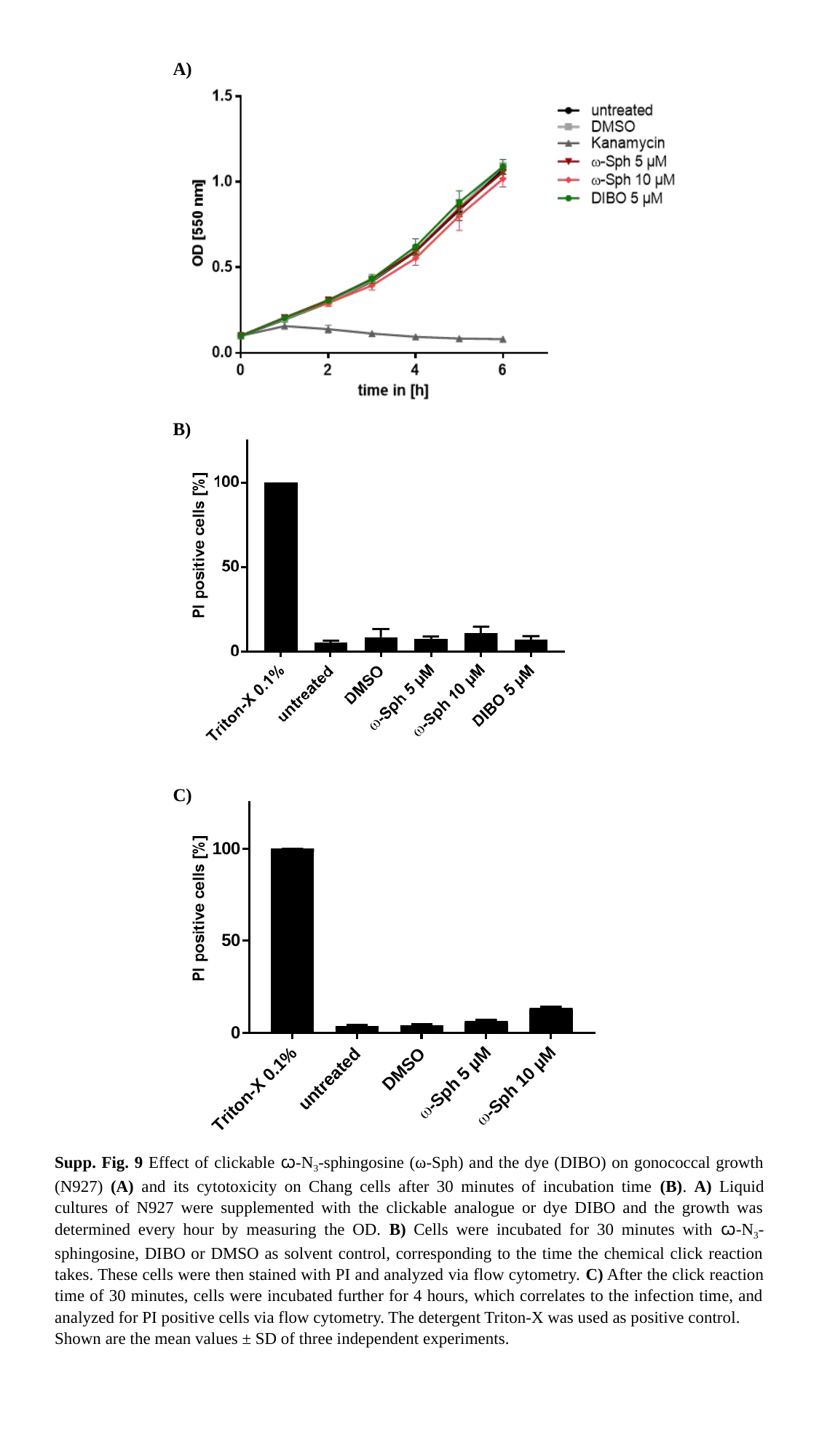

A)
B)
C)
Supp. Fig. 9 Effect of clickable ꙍ-N3-sphingosine (ω-Sph) and the dye (DIBO) on gonococcal growth (N927) (A) and its cytotoxicity on Chang cells after 30 minutes of incubation time (B). A) Liquid cultures of N927 were supplemented with the clickable analogue or dye DIBO and the growth was determined every hour by measuring the OD. B) Cells were incubated for 30 minutes with ꙍ-N3-sphingosine, DIBO or DMSO as solvent control, corresponding to the time the chemical click reaction takes. These cells were then stained with PI and analyzed via flow cytometry. C) After the click reaction time of 30 minutes, cells were incubated further for 4 hours, which correlates to the infection time, and analyzed for PI positive cells via flow cytometry. The detergent Triton-X was used as positive control.
Shown are the mean values ± SD of three independent experiments.

## Slide 10
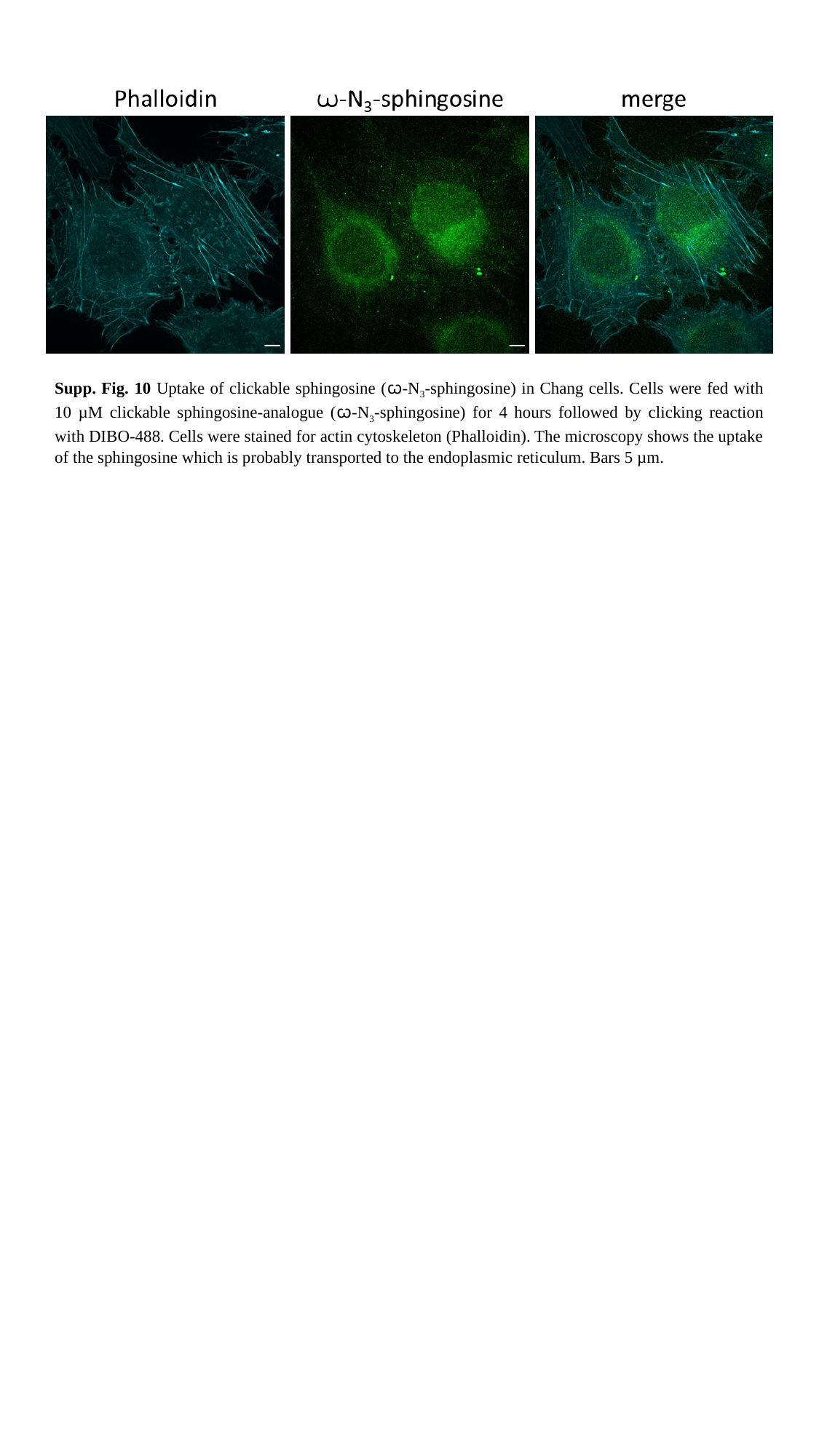

Supp. Fig. 10 Uptake of clickable sphingosine (ꙍ-N3-sphingosine) in Chang cells. Cells were fed with 10 µM clickable sphingosine-analogue (ꙍ-N3-sphingosine) for 4 hours followed by clicking reaction with DIBO-488. Cells were stained for actin cytoskeleton (Phalloidin). The microscopy shows the uptake of the sphingosine which is probably transported to the endoplasmic reticulum. Bars 5 µm.

## Slide 11
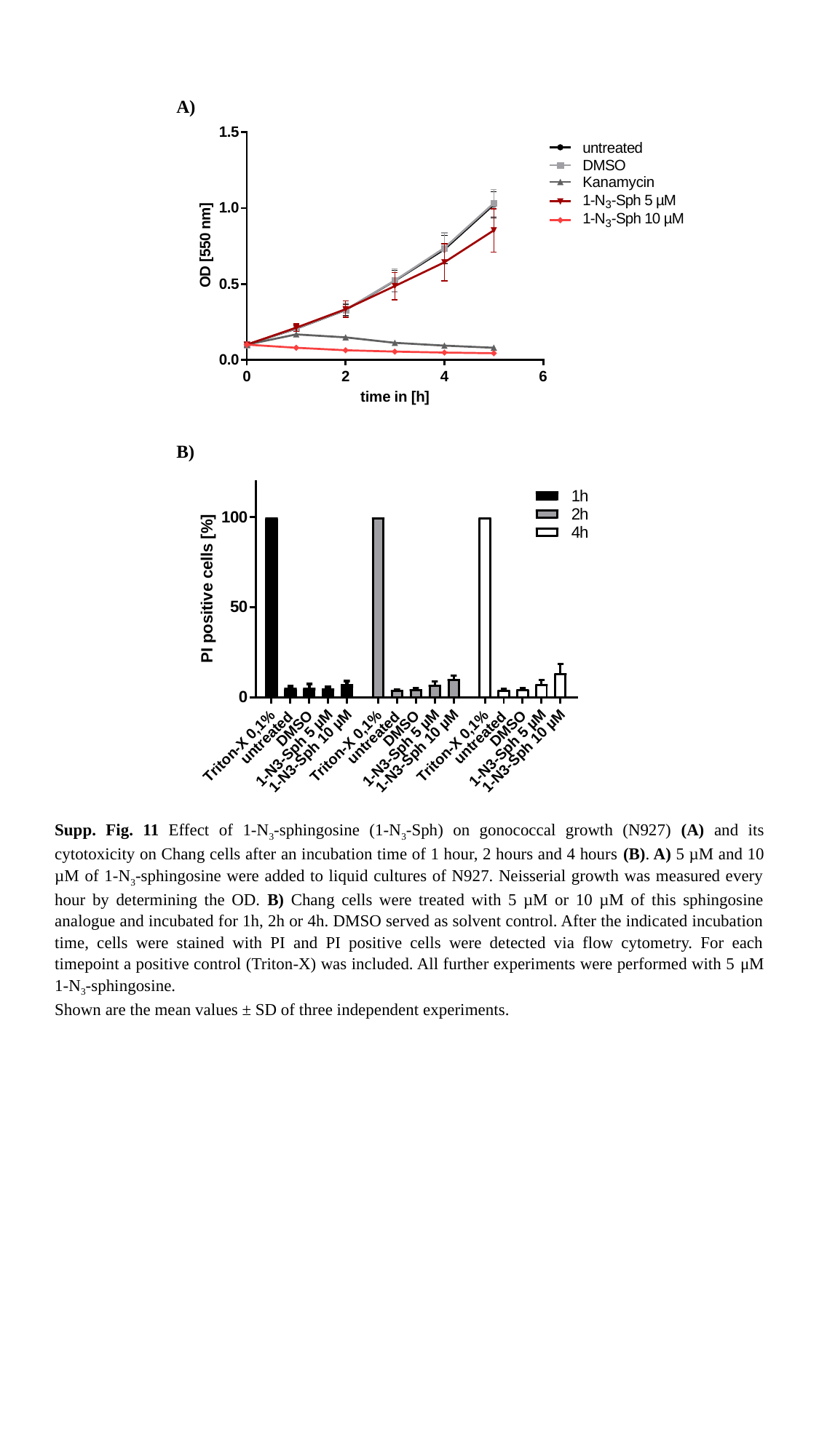

A)
B)
Supp. Fig. 11 Effect of 1-N3-sphingosine (1-N3-Sph) on gonococcal growth (N927) (A) and its cytotoxicity on Chang cells after an incubation time of 1 hour, 2 hours and 4 hours (B). A) 5 µM and 10 µM of 1-N3-sphingosine were added to liquid cultures of N927. Neisserial growth was measured every hour by determining the OD. B) Chang cells were treated with 5 µM or 10 µM of this sphingosine analogue and incubated for 1h, 2h or 4h. DMSO served as solvent control. After the indicated incubation time, cells were stained with PI and PI positive cells were detected via flow cytometry. For each timepoint a positive control (Triton-X) was included. All further experiments were performed with 5 μM 1-N3-sphingosine.
Shown are the mean values ± SD of three independent experiments.
